# A randomized controlled dismantling trial of post-workshop consultation strategies to increase effectiveness and fidelity to an evidence-based psychotherapy for Posttraumatic stress disorder

**DOI:** 10.1186/1748-5908-8-82

**Published:** 2013-08-01

**Authors:** Shannon Wiltsey Stirman, Norman Shields, Josh Deloriea, Meredith SH Landy, Jennifer M Belus, Marta M Maslej, Candice M Monson

**Affiliations:** 1 Women’s Health Sciences Division, National Center for PTSD, VA Boston Healthcare System, Boston University, 150 South Huntington Ave (116B3), Boston, MA 02130, USA; 2 Veterans Affairs Canada, Operational Stress Injuries National Network (OSINN), 305 Boul des Anciens-Combattants, Sainte-Anne-de-Bellevue, QC H9X 1Y9, Canada; 3 Department of Psychology, Ryerson University, 350 Victoria Street, Toronto, ON M5B 2K3, Canada; 4 Department of Psychology, University of North Carolina, 209 South Rd., Chapel Hill, NC 27599, USA

**Keywords:** Training, Implementation, Consultation, Psychotherapy, PTSD, Cognitive processing therapy

## Abstract

**Background:**

Posttraumatic Stress Disorder (PTSD) is a serious mental health condition with substantial costs to individuals and society. Among military veterans, the lifetime prevalence of PTSD has been estimated to be as high as 20%. Numerous research studies have demonstrated that short-term cognitive-behavioral psychotherapies, such as Cognitive Processing Therapy (CPT), lead to substantial and sustained improvements in PTSD symptoms. Despite known benefits, only a minority of clinicians provide these therapies. Transferring this research knowledge into clinical settings remains one of the largest hurdles to improving the health of veterans with PTSD. Attending a workshop alone is insufficient to promote adequate knowledge transfer and sustained skill; however, relatively little research has been conducted to identify effective post-training support strategies.

**Methods:**

The current study investigates whether clinicians receiving post-workshop support (six-month duration) will deliver CPT with greater fidelity (*i.e*., psychotherapy adherence and competence) and have improved patient outcomes compared with clinicians receiving no formal post-workshop support. The study conditions are: technology-enhanced group tele-consultation; standard group tele-consultation; and fidelity assessment with no consultation. The primary outcome is independent assessment (via audio-recordings) of the clinicians’ adherence and competence in delivering CPT. The secondary outcome is observed changes in patient symptoms during and following treatment as a function of clinician fidelity. Post-consultation interviews with clinicians will help identify facilitators and barriers to psychotherapy skill acquisition. The study results will inform how best to implement and transfer evidence-based psychotherapy (*e.g*., CPT) to clinical settings to attain comparable outcomes to those observed in research settings.

**Discussion:**

Findings will deepen our understanding of how much and what type of support is needed following a workshop to help clinicians become proficient in delivering a new protocol. Several influences on clinician learning and patient outcomes will be discussed. An evidence-based model of clinical consultation will be developed, with the ultimate goal of informing policy and influencing best practice in clinical consultation.

**Trial registration:**

ClinicalTrials.gov: NCT01861769

## Background

The lifetime prevalence of PTSD is approximately 9% in Canada’s general population [1], 6.8% in the United States (U.S.) [2], and 15% to 19% among veterans of the recent conflicts in Iraq and Afghanistan [3,4]. PTSD is associated with substantial functional impairment, suicide risk [5], numerous physical diseases [6-9], higher rates of mortality, high healthcare utilization and costs, and annual productivity loss of over $3 billion in the U.S. [10-15]. Studies of chronic PTSD indicate little change over time without active intervention [16].

Cognitive-behavioral therapies (CBT) for PTSD have been shown to be efficacious and are recommended as first-line therapies in treatment guidelines [17,18]. Cognitive Processing Therapy (CPT) is a 12-session, trauma-focused cognitive therapy that targets symptoms of PTSD and its comorbidities [19] that results in sustained, significant reductions in symptoms and increases in quality of life for diverse populations [20-34]. Despite substantial efficacy data, clinicians do not routinely deliver CPT or other evidence-based psychotherapies (EBPs) [35].

In Canada, the U.S., and the United Kingdom, policymakers have prioritized investigating the implementation of EBPs to address this critical gap between research and practice [36]. In response to the shortage of adequately trained clinicians [37], policymakers have issued mandates, provided incentives, and invested billions of dollars in EBP training of clinicians in public mental health settings [35,38]. However, many of the utilized training strategies are not empirically supported, and much remains to be learned about how best to disseminate and implement EBPs in routine clinical practice. Without proven strategies to increase clinician use, there is little assurance that the investment that individuals and organizations make to develop EBPs will ultimately benefit patients. Both the Canadian Institute of Health Research (CIHR)’s Health Research Roadmap (2009 to 2014; [39]) and the U.S. National Institute of Mental Health [40] note the growing need for implementation science to better understand the determinants and uptake of knowledge. The use of evidence-based implementation strategies will ensure that money spent on treatment research has maximum impact.

Didactic workshops are a cornerstone of existing large-scale EBP implementation efforts [35,38,41]. However, workshops alone are insufficient to promote high-quality implementation and sustainment of EBPs in clinical settings. After a single workshop, most clinicians are unable to deliver EBPs with fidelity (adherence to the protocol and competence in delivery of the interventions [42,43]). Fidelity to EBPs has been shown to predict patient outcomes for a number of mental health disorders [44-49], and has been associated with PTSD symptom outcomes in our preliminary research on CPT. To adequately prepare clinicians to deliver EBPs with fidelity, training packages that include post-workshop consultation and feedback have emerged as best practices, but little is understood about the relative benefits of different consultation strategies [41,42].

Previous comparisons of post-workshop consultation strategies have lacked external validity (*e.g*., have been conducted under controlled conditions with highly motivated therapists in training clinics), rarely assessed fidelity objectively and in real-world practice conditions [50], had poor follow-up rates, rarely addressed barriers to implementation, and have demonstrated less than optimal results [41-43]. Thus, there is little empirical evidence to identify optimal, cost-efficient consultation and feedback strategies. Feedback based on observation is considered the ‘gold standard’ for training and is assumed to be necessary for optimal fidelity [42]. Without more direct observation of therapist behavior, there is limited opportunity for the expert to provide the type of feedback required to promote fidelity and high-quality service delivery [51]. The one randomized study that tested this assumption found that coaching based on review of session material resulted in greater change talk among clients [52]. This study evaluated individual-level consultation under controlled conditions with limited external validity, and had no comparison to group consultation strategies. Individual feedback and consultation is too costly and resource-intensive to be feasible in large healthcare delivery systems [53]. One strategy used in several training initiatives is expert-delivered group tele-consultation, in which clinicians receive feedback from an expert on their treatment provision based solely on their verbal reports of their sessions [35]. However, the association between clinician report of their behavior and objective assessment of therapy fidelity is unknown.

To address this limitation of the standard consultation strategy, the present study also tests a technology-enhanced group tele-consultation. Our approach involves an expert and group members listening to, and providing feedback on, portions of group members’ audio-recorded therapy sessions on a weekly basis. This strategy provides exposure to a broad sample of case material for vicarious learning, and also allows the expert to provide more specific and accurate feedback than in standard consultation. However, while it provides the opportunity for more in-depth discussion of work samples for the clinicians who present in a given week, the time spent on the audio review may result in less time for other clinicians in the group to discuss their own cases when not presenting audio. Thus, each consultation strategy may have unique advantages over the other. The two consultation conditions will be compared to a no consultation (fidelity monitoring only) condition in order to assess the incremental benefit of post-workshop consultation. The study is funded by the Canadian Institutes of Health Research and represents the collaborative efforts of investigators at Ryerson University, Veterans Affairs Canada’s Operational Stress Injury National Network (OSINN), the U.S. National Center for PTSD, and Boston University. This article describes the study’s aims, method and proposed analytic strategy.

### Research questions and hypotheses

Question 1: The principal research question to be addressed is whether different types of post-workshop support for mental health clinicians have differential effects on clinician fidelity to the CPT protocol.

Hypothesis 1: We hypothesize that the technology-enhanced group tele-consultation condition will evidence the highest levels of fidelity, the standard group tele-consultation condition will evidence intermediate levels of fidelity, and the no consultation condition will evidence the lowest fidelity.

Question 2: Is clinician fidelity to the CPT protocol is related to client outcomes?

Hypothesis 2: We hypothesize that fidelity to the CPT protocol, irrespective of consultation condition, will be positively associated with improved client outcomes.

Question 3: What are contextual barriers or facilitators to the skilled use of CPT in their routine practice?

Hypothesis 3: We hypothesize that organizational contextual variables such as local leadership support, organizational structure, and organizational climate, as well as clinician attitudes toward EBPs will influence the uptake of CPT.

## Methods

This randomized controlled dismantling trial has independent fidelity rating as the primary outcome and patient outcomes as the secondary outcomes. Clinicians are randomly assigned to six months of post-workshop support following an intensive two-day CPT workshop delivered by the last author. The conditions are: standard group tele-consultation, technology-enhanced group tele-consultation, or no consultation/fidelity monitoring only. The post-workshop consultation conditions vary as to whether audio-recorded therapy sessions and other work samples, such as CPT worksheets, are reviewed in the group. This design permits the evaluation of the additive value of each of the elements (*i.e*., expert consultation, technology-assisted expert consultation). Follow-up assessments of fidelity will be conducted at six months post-workshop for clinicians, and follow-up symptom assessments will be conducted at three months post-treatment for patients.

Participation in the study requires that all clinicians consent at least two clients to have all of their CPT sessions audio-recorded for use in consultation and/or fidelity rating. Four therapy sessions will be randomly selected across the six-month period for fidelity rating, and fidelity to treatment will be assessed by trained graduate students and post-doctoral fidelity assessors. Consistent with recommended clinical practice and research trials, patient outcomes will be assessed pre-treatment, at alternate therapy sessions, and at three months post-treatment. A secure web-based application is used to manage the continuous uploading of encrypted audio-recorded therapy sessions to the study server and to protect the privacy and confidentiality of patients and clinicians. Study-provided digital audio-recorders (USB-equipped) are used to record, store and upload CPT sessions to the secure server for fidelity ratings. Study staff retrieve the randomly-selected audio-recordings and assign them to the CPT fidelity raters, who are blinded to study condition. To check if rater blinding has been achieved, fidelity raters will be asked to guess the study condition for each session they rated. Patient self-report data is collected by the clinicians, who then upload the data directly to the server and clinician CPT activity outside of study-related procedures is also recorded.

### Interventions

Participants in all conditions attend the workshop, receive the CPT manual and related materials, and have access to resources available through the free CPT-web online training (https://cpt.musc.edu/index). All participants who upload the required number of session recordings and patient symptom measures will be eligible to receive ‘CPT Provider’ status. This status is not tied to any change in remuneration for services. Provider status will be awarded to clinicians who demonstrate an acceptable level of fidelity to CPT based on session recordings randomly selected from the last two weeks of the six-month post-workshop period. Workshop participants who provide informed consent are randomized to one of the three post-workshop consultation conditions after completing the workshop. Participants in the two tele-consultation conditions receive six months of weekly one-hour group tele-consultation with a CPT expert. Each call includes discussions of the CPT protocol, challenging cases, treatment obstacles, and specific issues raised by participants within each group. Both tele-consultation conditions include a maximum of six to eight clinicians per call. CPT experts provide consultation across both consultation conditions to control for any expert-related effects. The prescribed and proscribed activities for each consultation condition are described in manuals, and study team members (SWS, CMM, ML) review consultant calls on a monthly basis to ensure that fidelity to the consultation condition is maintained. Consultants are trained by study team members prior to beginning consultation calls and receive consultation and feedback every four to six weeks or as needed for the first two waves of consultation, and as needed during the third consultation wave.

### Inclusion and exclusion criteria

Mental health clinicians from the Operational Stress Injury clinics, Canadian Forces mental health services, and the broader Canadian community are eligible to participate in the study if they: attend a CPT workshop; are licensed as a mental health clinician and are able to practice independently; currently provide psychotherapy to patients with PTSD; consent to be randomized to one of the study conditions; and are willing to solicit patient participation. Eligible patient participants are: diagnosed with PTSD; and consent to have their sessions audio-recorded and listened to by a fidelity rater and potentially other clinicians teaching and learning CPT. Patients are permitted to continue other psychotherapeutic interventions if they are not specifically focused on treating PTSD symptoms. Ineligible patients include those not eligible for CPT based on the state of research evidence, including those with: current uncontrolled psychotic or bipolar disorder; substance dependence; imminent suicidality or homicidality that requires acute care; and significant cognitive impairment (although mild to moderate traumatic brain injury is permitted).

### Outcome measures and assessment procedures

#### Fidelity ratings and assessment of CPT modification from audio-recorded CPT sessions

This study uses a modified version of the CPT fidelity measure [54] that has been used in previous clinical trials [55]. The CPT fidelity measure examines clinicians’ adherence to, and competence in, delivering specific CPT interventions prescribed in each session. Clinicians are rated on their adherence and competence in delivering these elements (rated on a 7-point, Likert-type scale). Two studies have assessed the reliability of the measure and found 97% agreement between two raters across all items for adherence in one study (agreement was not reported for competence scores; [55]) and 100% agreement for adherence and overall competence ratings in another [56]. Pilot research has been conducted to assess the predictive validity of the unique and essential scales for a small sample of veterans. CPT competence was highly correlated across all sessions with competence in cognitive therapy as measured by a validated cognitive therapy fidelity-assessment instrument, r = 0.82, p = 0.001 [44,57]. Therapist competence in the first session of CPT was positively correlated with self-reported symptom improvement among patients from baseline to post-treatment, r = 0.79, p = 0.02. Additionally, we use a framework of modifications to identify any deviations from or adaptations to CPT that occur in the sessions [58]. At least 10% of the sessions will be rated by all raters to independently assess inter-rater agreement.

### Patient outcome measures

Patients are asked to complete measures related to their PTSD symptoms, functional impairment and overall quality of life at baseline (pre-treatment), at every alternate CPT treatment session, and at post-treatment follow-up (three months post-treatment). The assessment measures include: ‘The Posttraumatic Stress Disorder Checklist’ (PCL-S; [59]), a 17-item questionnaire that reliably measures the severity of distress related to PTSD symptoms (Chronbach’s alpha = 0.94). Each item is measured on a 5-point Likert scale, which maps on to the DSM-IV criteria for PTSD; ‘The Outcome Questionnaire – 45’ (OQ- 45; [60]), a 45-item self-report measure of three dimensions of client functioning in the past week: symptom distress (*e.g.*, anxiety, depression), interpersonal problems, and social role adjustment (Chronbach’s alpha = 0.71 to 0.93). The OQ-45 has established clinical cut-offs and a reliable change indices to signal meaningful improvements or deterioration; and ‘The Short-Form Quality of Life Health Survey’ (SF-12; [61]), a 12-item survey that measures mental components (vitality, social functioning, role-emotional, and mental health) and physical components (physical functioning, role-physical, bodily pain, and general health) of health related to quality of life. All questionnaires are available in English and French, and take clients around 15 to 20 minutes to complete. Therapists upload summary scores to a secure website at the same time that they upload their audio-recordings.

### Clinician demographic characteristics and experience

A pre-workshop questionnaire is administered to assess relevant clinician demographic information (*i.e*., age, gender, education), clinician experience (years of licensed practice, hours of formal training in CBT, hours of supervised post-graduate CBT training), prior CPT training experience (workshop hours and supervision hours), experience treating clients with PTSD (number of clients), and volume of current clinical activity (caseload and hours devoted to psychotherapy).

### Clinician monthly CPT activity reporting

During the six-month active study period, all clinicians complete a monthly report via a web-based survey on CPT activity. Questions include: the number of CPT sessions provided over the past month (irrespective of patients’ enrollment in the study); cumulative number of new CPT clients seen; frequency, duration, and satisfaction with consultation over the past month (as applicable); and a rating of clinician confidence in their ability to adhere to adherence to the CPT protocol.

### Quantitative and qualitative measurement of potential barriers and facilitators to CPT skills uptake and implementation outcomes

The Organizational Readiness to Change Questionnaire [62] is a pre-workshop self-report questionnaire that assesses various elements of a clinician’s perceived support for training implementation. A total of 18 scales (Chronbach alphas = 0.56 to 0.92) are grouped within four dimensions of readiness (*i.e*., motivation for change, adequacy of resources, staff attributes, organizational climate). The Dimensions of Organizational Readiness-Revised (DOOR-R; [63]) scale assesses perspectives on intra- and extra-organizational implementation readiness. The Evidence-Based Practice Attitudes-50 (EBPAS-50; [64]) is a 50-item, self-report measure of clinician attitudes toward EBPs, consisting of 12 factors, with moderate to large factor loadings and fair to excellent internal consistency reliabilities.

We also interview clinicians at baseline, post-consultation, and three months post-consultation to further assess for barriers and facilitators to the use of CPT in their practice. The interviews query effective tactics and methods that may have been used outside of the consultation methods (*i.e*., positive deviance; [65]) that the clinicians deemed helpful in acquiring and maintaining CPT skills. We based our interview questions on those used to assess influences on training and implementation in previous research [66], which are based in part on the Consolidated Framework for Implementation Research (CFIR [67]).

### Sample size and justification for power calculations

In conducting power calculations, we considered assessment attrition, within-clinician correlation, and within-setting effects. Based on a power curve calculation with the following assumptions: 30 clinicians per condition; 4 sessions rated for fidelity per clinician, randomly selected over time; with an alpha-level of 0.05, within provider correlation estimated at 0.38, 10% proportion of variance accounted for by the random effect associated with agency, based on a two-sided test, the detectable effect size for at least 80% power will be medium to large, per Cohen’s [68] classifications, ranging from d = 0.41 to 0.55.

### Recruitment and randomization

We recruited OSI clinicians directly. Recruitment took place at CPT workshops in Canada from 2012 to 2013. Three months before the first workshop, the OSINN invited clinicians and clinic coordinators from the OSI clinic network, Canadian Forces health services, and approximately 800 community-based clinicians who provide services to veterans with PTSD. The clinicians include psychologists, social workers, psychiatric nurses, and psychiatrists. This invitation was accompanied by a letter soliciting participation in the study. Prior to enrolling clinicians, ethics approval for the study was obtained from the clinic or institution where the clinician worked. Community clinicians were informed that the CHIR-supported workshop was free, and 24 clinicians received up to $500 reimbursing travel and accommodation. Partial reimbursement for the community clinicians was intended to offset the barrier of cost associated with a two-day workshop. Following the initial CIHR-funded workshop, two provincial health authorities and one hospital hosted workshops. One of the authors (CMM) was the primary CPT trainer. Interested clinicians consented prior to, or within the week following the workshop. Consent forms outlined the purpose and requirements of the study, as well as potential risks. A random allotment of participants was generated by the study statistician for each condition. To avoid any biases in condition assignment, condition assignment was communicated by participant number to the study coordinator after participant numbers had been assigned to the consented participants. Clinicians received a notification e-mail from the study coordinator within one week after the workshop regarding their randomized condition. The e-mail also included consultant contact information and a schedule of consultation sessions (if applicable to their condition). As in other studies of this nature (e.g., [52]), the clinicians were asked to provide an information sheet about the study and obtain verbal consent from patients who agreed to participate in the study, then document patient consent in his/her medical records. Upon returning to their respective work places, participants began audio-recording CPT sessions with consented patients.

### Proposed analytic strategy

Trial reporting will follow the CONSORT 2010 statement for reporting randomized controlled trials [69]. Analyses will be preceded by careful examination of the descriptive statistics and distributional form of all variables. The effectiveness of randomization will be evaluated by comparing the different conditions on baseline measures, demographics, and key outcome variables using Chi-squared tests and t-tests. The primary analyses will be conducted using random effects regression modeling. These models provide more flexibility in modeling missing data than general linear models and are appropriate for nested data. ‘Intention-to-train’ principles will be used in the analyses of clinicians who are assigned to a consultation modality but either fail to engage or are unable to fulfill the post-workshop participation requirements (*e.g*., do not attend consultation sessions). Analyses of both the intention-to-train and completer samples will be conducted.

To explore the contribution of organization and clinician-level factors to the outcomes of interest, in addition to random effects regression modeling, we will conduct qualitative analyses on interview data. Qualitative and quantitative data will be integrated to facilitate a fine-grained understanding of processes and characteristics that influence training outcomes. Interviews will be analyzed in line with goals, objectives and key research questions. Using content analysis, we will identify analytical categories to describe and explain our observations. Our work will occur in five stages outlined in Pope, Ziebland, and Mays’ framework approach to qualitative analysis [70]: Familiarization; Identifying a Thematic Framework; Indexing; Charting; and Mapping and Interpretation. In the second stage, codes will be derived deductively by identifying categories at the beginning of the research (*e.g*., elements of the CFIR), and inductively by identifying those that emerge gradually from the data. In subsequent phases, survey data on contextual factors will be examined in conjunction with fidelity scores for the purposes of validation, hypothesis generation, and expansion [71,72] to understand ways in which contextual factors influence fidelity to CPT.

### Trial status

To date, six workshops have been completed, and, through efforts to enroll clinicians over three waves of recruitment, the recruitment goal of 90 clinicians was exceeded. The first and second waves of consultation have been completed, and the third wave is underway. Pre- and post-training interviews with providers from waves one and two are complete. Thus far, our retention rate and compliance with study procedures (*e.g*., uploading of sessions) has been high, and a high proportion of clinicians uploaded recordings at baseline and over the course of the consultation period. We anticipate that data collection will be complete in early 2014.

## Discussion

There are four significant innovations that the current project offers the field of Implementation Science. First, this will be the first study to examine psychotherapy training and consultation within a broader implementation context. Previous randomized training studies have not examined the organizational context. Our use of mixed-methods research strategies in the context of a randomized controlled trial [72] will allow us to examine a broad range of potential influences on the outcomes that we are assessing. This approach will extend our understanding of interactions between these factors and training outcomes.

A second major advancement is the empirical investigation of the optimal (efficacious, scalable and replicable) methods of post-workshop consultation. To adequately test the incremental value of consultation, we are conducting a randomized, dismantling research design, which includes manipulation of the type of post-workshop consultation while holding other variables constant. Results will help inform policy on post-workshop support. The inclusion of the fidelity monitoring only condition allows for testing an as yet unevaluated and potentially efficient strategy for increasing fidelity to EBPs: recording sessions with the knowledge that they will be reviewed and evaluated for fidelity. Our control condition allows for the formal testing of this possibility while allowing us to examine the incremental value of different types of expert consultation.

Although fidelity is a key implementation outcome in the model by Proctor and colleagues [73] and in this study, fidelity to EBPs has not been adequately assessed by independent evaluators in prior implementation research. It has either not been assessed at all, or has been assessed through self-report, standardized patient protocols [74], or a single session selected by providers (c.f., [52,75]). Our third innovation is to improve upon this methodological limitation of prior studies by randomly selecting audio-recorded sessions for fidelity monitoring. We will also conduct the only investigation of the effect of clinician fidelity to an EBP in routine care settings on PTSD outcomes to date. Because this investigation will examine sessions from novice clinicians who received varying intensities of consultation, we expect to see a greater range of fidelity than that seen in previous investigations of the impact of fidelity on symptom outcomes, as most studies to date have used clinical trial data with sessions from closely supervised clinicians. Finally, no evidence-based guidance exists regarding how to facilitate fidelity to the CPT protocol and ultimately patient outcomes. Using transcripts from consultation sessions, members of our research team will develop and test a theory of evidence-based consultation.

### Ethical

The study has been approved by the Ryerson University Research Ethics Board, and the Boston VA and Boston University Institutional Review Boards.

## Abbreviations

PTSD: Posttraumatic stress disorder; CPT: Cognitive processing therapy; CBT: Cognitive behavioral therapy; EBP: Evidence based psychotherapy.

## Competing interests

The authors declare that they have no competing interests.

## Authors’ contributions

CMM, SWS, and NS designed the study and each wrote sections of the proposal. ML, JD, MMM, and JB provided a critical review of the manuscript, contributed to the development and description of study procedures and compiled information on the current status of the research. All authors had the opportunity to review the final draft of this manuscript.
